# Novel anthropometric indices for predicting type 2 diabetes mellitus

**DOI:** 10.1186/s12889-024-18541-7

**Published:** 2024-04-13

**Authors:** Erfan Sadeghi, Alireza Khodadadiyan, Seyed Ali Hosseini, Sayed Mohsen Hosseini, Ashraf Aminorroaya, Massoud Amini, Sara Javadi

**Affiliations:** 1 Department of Biostatistics, School of Medicine, Shiraz University of Medical Sciences, Shiraz, Iran; 2 Department of Cardiovascular Research Centre, Shiraz University of Medical Sciences, Shiraz, Iran; 3 School of Medicine, Shiraz University of Medical Sciences, Shiraz, Iran; 4 Department of Biostatistics & Epidemiology, School of Public Health, Isfahan University of Medical Sciences, Isfahan, Iran; 5 Isfahan Endocrine and Metabolism Research Center, Isfahan University of Medical Sciences, Isfahan, Iran; 6 Shiraz University of Medical Sciences, Shiraz, Iran

**Keywords:** Anthropometric indices, Diabetes mellitus, Diagnosis, Waist-to-hip ratio, Waist-to-height ratio, Body roundness index, A body shape index, Visceral adiposity index, Body mass index, Body adiposity index

## Abstract

**Background:**

This study aimed to compare anthropometric indices to predict type 2 diabetes mellitus (T2DM) among first-degree relatives of diabetic patients in the Iranian community.

**Methods:**

In this study, information on 3483 first-degree relatives (FDRs) of diabetic patients was extracted from the database of the Endocrinology and Metabolism Research Center of Isfahan University of Medical Sciences. Overall, 2082 FDRs were included in the analyses. A logistic regression model was used to evaluate the association between anthropometric indices and the odds of having diabetes. Furthermore, a receiver operating characteristic (ROC) curve was applied to estimate the optimal cutoff point based on the sensitivity and specificity of each index. In addition, the indices were compared based on the area under the curve (AUC).

**Results:**

The overall prevalence of diabetes was 15.3%. The optimal cutoff points for anthropometric measures among men were 25.09 for body mass index (BMI) (AUC = 0.573), 0.52 for waist-to-height ratio (WHtR) (AUC = 0.648), 0.91 for waist-to-hip ratio (WHR) (AUC = 0.654), 0.08 for a body shape index (ABSI) (AUC = 0.599), 3.92 for body roundness index (BRI) (AUC = 0.648), 27.27 for body adiposity index (BAI) (AUC = 0.590), and 8 for visceral adiposity index (VAI) (AUC = 0.596). The optimal cutoff points for anthropometric indices were 28.75 for BMI (AUC = 0.610), 0.55 for the WHtR (AUC = 0.685), 0.80 for the WHR (AUC = 0.687), 0.07 for the ABSI (AUC = 0.669), 4.34 for the BRI (AUC = 0.685), 39.95 for the BAI (AUC = 0.583), and 6.15 for the VAI (AUC = 0.658). The WHR, WHTR, and BRI were revealed to have fair AUC values and were relatively greater than the other indices for both men and women. Furthermore, in women, the ABSI and VAI also had fair AUCs. However, BMI and the BAI had the lowest AUC values among the indices in both sexes.

**Conclusion:**

The WHtR, BRI, VAI, and WHR outperformed other anthropometric indices in predicting T2DM in first-degree relatives (FDRs) of diabetic patients. However, further investigations in different populations may need to be implemented to justify their widespread adoption in clinical practice.

## Background

In recent decades, T2DM has increasingly become a significant public health issue globally, especially in the past few decades. The prevalence of T2DM has increased to 11.6% globally, impacting a population of more than 100 million adults [1]. One of the most important risk factors for T2DM is obesity. There is a growing recognition that obesity is a modifiable risk factor for prediabetes, and T2DM has various aspects according to its extent, pattern, timing, and duration [2]. Moreover, not only are FDRs of individuals with diabetes at greater risk than second-degree relatives, but they also exhibit increased whole-body insulin resistance and decreased muscle glucose uptake [3]. In epidemiological studies, anthropometric indices have been utilized to measure obesity because of their simplicity and utility [4].

Classic anthropometric indices include BMI, waist circumference (WC), waist-to-hip ratio (WHR), and waist-to-height ratio (WHtR) [4–6]. BMI is a simple index of weight-to-height that is commonly used to classify overweight and obesity in adults [7]. Studies have shown that BMI is not able to distinguish muscle tissue from fat accumulation, so it cannot reflect abdominal fat. Recently, BMI has been criticized because it does not accurately measure body weight and fat directly but relies on body weight alone [8]. Among traditional anthropometric indices, the WHR and WHtR are indices of central obesity and are correlated with visceral body fat [9]. In addition, abdominal obesity was measured by waist circumference (WC). According to the study by Jamar et al., WHtR predicts insulin resistance more precisely than WC or BMI [10]. Furthermore, based on analyses from similar studies, optimal cutoff values of the WHtR were used to predict diabetes [11, 12]. However, some published studies have reported BMI or WC as the best predictors of diabetes [4, 13–15].

Novel indices, such as the body shape index (ABSI), body roundness index (BRI), and visceral adiposity index (VAI), have been proposed as alternative indicators of obesity [4]. The ABSI is a new anthropometric index based on normalizing WC to BMI and height [16]. According to the literature, the ABSI, which is independent of BMI by design, provides efficient risk stratification for underweight and obese individuals. However, we are not sure whether the ABSI could also predict the new onset of diabetes mellitus (DM) in our population [17]. The BRI is a potential alternative measure for evaluating obesity in individuals with T2DM [4]. In addition, the BRI is an indicator of obesity and is based on body fat (BF) and body fat percentage (BF%) [18]. This index is closely associated with diabetes risk and was used to identify diabetes in a cross-sectional study [19, 20]. According to one study, BRI can predict development of diabetes based on height, weight, waist circumference, and hip circumference [4].

Due to the difficulties of assessing BMI at the nutritional level and its limited accuracy, Bergman et al. developed the body adiposity index (BAI) for adults as an alternative new parameter for evaluating body composition based on height in meters and hip circumference in centimeters [21]. Bozorgmanesh et al. reported that the VAI, an indicator of visceral fat dysfunction, has good predictive performance for diabetes in Iran [22] and is also a sex-specific index that indirectly reflects visceral adipose function [23, 24]. Another study has shown that the VAI is a good predictor of T2DM [25]. Cutoff points for anthropometric indices such as the BRI, BAI, and VAI are not unified among different populations [26–28]. However, no comprehensive agreement has been reached on the best anthropometric index for predicting the development of T2DM in FDRs of diabetic patients. The present study aimed to compare anthropometric indices for predicting T2DM among first-degree relatives of diabetic patients in the Iranian community.

## Methods

### Study participants

In this study, baseline information on 3483 FDRs of diabetic patients was extracted from the database of the Endocrinology and Metabolism Research Center of Isfahan University of Medical Sciences, known as the Isfahan Diabetes Prevention Study (IDPS), the details of which have been presented elsewhere [29, 30]. In summary, the IDPS is an ongoing longitudinal study initiated between 2003 and 2005 in Isfahan, central Iran. The primary aim of this study was to examine the potential risk factors for diabetes in individuals with a family history of T2DM. During the evaluations, participants underwent physical measurements and laboratory tests, including a standard 75-g, 2-hour oral glucose tolerance test (OGTT). Diabetes status was defined as having a fasting plasma glucose (FPG) level equal to or higher than 126 mg/dL, a 2-hour plasma glucose level equal to or higher than 200 mg/dL, or a HbA1c level equal to or higher than 6.5%. Normal status was defined as having an FPG level below 100 mg/dL, a 2-hour plasma glucose level below 140 mg/dL, or an HbA1c level below 6.0%. The participants also completed a questionnaire on their health status and various factors potentially associated with the risk of diabetes. Follow-up assessments adhered to standard medical care for diabetes [31], focusing on gathering updated information on demographics, physical measurements, lifestyle factors, and newly diagnosed diabetes cases. Participants with a normal baseline OGTT result underwent repeat testing at least every 3 years, while those with abnormal results usually underwent annual repeat testing. The inclusion criteria were siblings and children of type 2 diabetes patients aged 30 to 70 years. We excluded participants who had a prediabetic baseline status defined as impaired fasting glucose (IFG) (FPG: 100–125 mg/dL and 2-h plasma glucose < 140 mg/dL) or impaired fasting glucose (IGT) (FPG < 126 mg/dL, but with 2-h plasma glucose concentration ≥ 140 and < 200 mg/dL) or HbA1c 6.0–6.49% [32] or were missing data, resulting in the exclusion of 1401 participants. All participants signed informed written consent for their participation. The present study was conducted based on the principles of the Declaration of Helsinki and the approval of the ethics committee of Isfahan University of Medical Sciences.

### Measurements

The participants’ height and weight were measured in light clothing using a Seca weighting scales and stadiometer. The BMI was calculated by dividing weight in kilogram (kg) by height squared in meter (m2) [33]. To measure waist circumference (WC), the midpoint between the lowest point of the rib and the top edge of the iliac crest was measured [34]. Hip circumference (HC) was utilized to quantify the horizontal extent or placement of the hip protrusion. Tape measures were used to measure WC and HC to the nearest 0.1 cm [35]. The WHR and WHtR were calculated as WC divided by HC and WC divided by height, respectively [36–38].

Other indices were calculated using the following formulas:

**Table:** 

Index	Reference
$$ABSI=\frac{WC}{BMI^{2}/_{3}\ {height}^{1}/_{2}}$$	[39]
$$BRI=364.2-365.5\times \sqrt{1-\frac{{\left(\frac{WC}{2\pi}\right)}^2}{{\left(0.5\ height\right)}^2}}$$	[39]
$$BAI=\frac{HC}{height^{1.5}}-18$$	[21]
$$VAI\ (Men)=\left(\frac{WC(cm)}{39.68+\left(1.88\times BMI\right)}\right)\times \left(\frac{TG\left(\frac{mmol}{Ll}\right)}{1.03}\right)\times \left(\frac{1.31}{HDL-c\left(\frac{mmol}{L}\right)}\right)$$	[40]
$$VAI\ (Women)=\left(\frac{WC(cm)}{39.58+\left(1.89\times BMI\right)}\right)\times \left(\frac{TG\left(\frac{mmol}{Ll}\right)}{0.81}\right)\times \left(\frac{1.52}{HDL-c\left(\frac{mmol}{L}\right)}\right)$$	[40]

### Statistical analysis

Anthropometric indices are presented as the mean (standard deviation) and were compared between diabetic patients and nondiabetic patients using Student’s t test. Due to the differences in the scale of the indices, we standardized them so that we could easily compare their effects. Therefore, we first computed the sample mean and standard deviation of the indices separately for all males and females. Then, z-scores were calculated as follow: (measurement value—mean) / standard deviation. The association of T2DM risk and anthropometric indices were examined using univariate logistic regression with T2DM status as the binary dependent variable, and anthropometric indices as the independent variables. Moreover, a receiver operating characteristic (ROC) curve analysis was performed to estimate the diagnostic parameters to compare the discrimination ability of the anthropometric indices, and to determine the optimal cutoff points of the indices based on the Youden index. The Statistical Packages for Social Sciences (SPSS) version 24 and MedCalc version 20.104 were used for data analysis. *P* values < 0.05 were considered to indicate statistical significance.

## Results

A total of 2082 FDR subjects, ranging from 30 to 70 years old, were included in the present study, of whom 318 (15.3%) had diabetes (103 male and 215 female). The mean age of the males was 43.17 ± 7.20 years, while that of the females was 43.18 ± 6.10 years. For both the male and female groups, Table 1 shows that the mean values of almost all indices were significantly greater in the T2DM group than in the normal control group (*P* < 0.05). The logistic regression model revealed that all of the indices were significantly associated with increased risk of T2DM; for instance, each one-unit increase in BMI z-score was associated with increased the risk of T2DM by 33% in males (OR = 1.33, 95% CI = [1.07, 1.64], *P* = 0.008) and each one-unit increase in the WHtR z-score was associated with increased the risk of T2DM by 90% (OR = 1.90, 95% CI = [1.64, 2.20], *P* < 0.001) in females (Table 2).

**Table 1:** Descriptive information of the anthropometric indices

Gender	Index	Total M (SD)	T2DM M (SD)	Non-T2DM M (SD)	***P*** value
**Male**	BMI	27.35 (3.68)	28.23 (3.58)	27.15 (3.67)	0.007
	WHtR	0.55 (0.05)	0.57 (0.05)	0.54 (0.05)	< 0.001
	WHR	0.90 (0.06)	0.93 (0.05)	0.90 (0.05)	< 0.001
	ABSI	0.08 (0.01)	0.079 (0.01)	0.08 (0.01)	< 0.001
	BRI	4.46 (1.11)	4.93 (1.08)	4.35 (1.08)	< 0.001
	BAI	28.83 (6.12)	29.73 (3.93)	28.61 (3.42)	0.005
	VAI	6.69 (4.94)	7.80 (5.39)	6.42 (4.81)	< 0.001
**Female**	BMI	29.14 (4.49)	30.68 (4.71)	28.89 (4.40)	< 0.001
	WHtR	0.56 (0.06)	0.59 (0.06)	0.55 (0.061)	< 0.001
	WHR	0.80 (0.06)	0.83 (0.05)	0.79 (0.05)	< 0.001
	ABSI	0.073 (0.01)	0.075 (0.01)	0.073 (0.01)	< 0.001
	BRI	4.56 (1.37)	5.35 (1.54)	4.42 (1.30)	< 0.001
	BAI	67.49 (5.16)	39.04 (5.93)	37.23 (4.98)	< 0.001
	VAI	6.18 (4.95)	7.82 (4.71)	5.92 (4.93)	< 0.001

M mean, SD standard deviation

*BMI* Body mass index, *WC* Waist circumference, *WHtR* Waist-to-height ratio, *WHR* Waist-to-hip ratio, *ABSI* A body shape index, *BRI* Body boundness index, *BAI* body adiposity index, and *VAI* Visceral adiposity index

**Table 2:** The results of univariate logistic regression for evaluating the association of T2DM risk and z-scores of the anthropometric indices

Indices	Male		Female	
	OR (95% CI)	*P* value	OR (95% CI)	***P*** value
BMI z-score	1.33 (1.07, 1.64)	0.008	1.67 (1.27, 1.67)	< 0.001
WHtR z-score	1.76 (1.38, 2.23)	< 0.001	1.90 (1.64, 2.20)	< 0.001
WHR z-score	1.87 (1.43, 2.43)	< 0.001	1.84 (1.59, 2.14)	< 0.001
ABSI z-score	1.50 (1.18, 1.91)	0.001	1.82 (1.56, 2.11)	< 0.001
BRI z-score	1.70 (1.35, 2.14)	< 0.001	1.82 (1.58, 2.10)	< 0.001
BAI z-score	1.36 (1.09, 1.68)	0.005	1.39 (1.21, 1.60)	< 0.001
VAI z-score	1.27 (1.04, 1.55)	0.020	1.34 (1.18, 1.53)	< 0.001

*BMI* Body mass index, *WC* Waist circumference, *WHtR* Waist-to-height ratio, *WHR* waist-to-hip ratio, *ABSI* A body shape index, *BRI* Body boundness index, *BAI* Body adiposity index, *VAI* Visceral adiposity index, *OR* odds ratio, and *CI* confidence interval

**Fig. 1:**
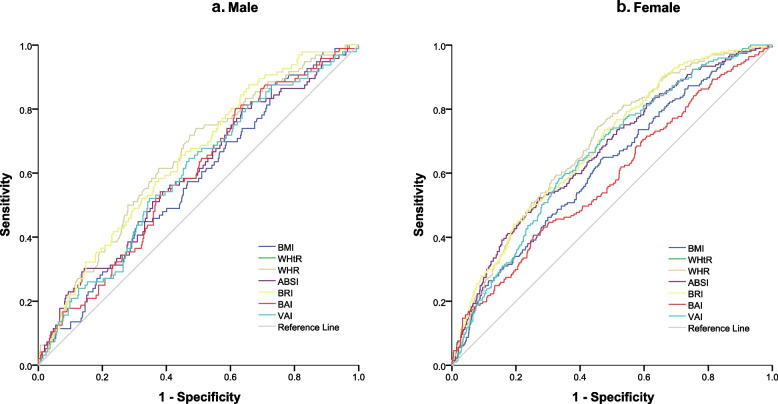
ROC curve for the anthropometric indices in male (**a**) and female (**b**)

Figure 1 presents the ROC curves for the anthropometric indices of men and women. Table 3 lists the diagnostic parameters, including the sensitivity, specificity, optimal cutoff values, *P* value, and area under the curve (AUC), of the anthropometric indices for predicting T2DM according to sex. Furthermore, in women, the area under the curve (AUC) values of all the incidences were significantly greater than those in men. Table 3 presents the associations between z-scores for various anthropometric indices (namely, BMI, WHR, WHtR, ABSI, BAI, BRI, and VAI) and risk of diabetes. According to the confidence intervals in Table 3, the WHR, WHtR, and BRI were the strongest predictors of T2DM risk in both the male and female groups. BMI and BAI were the weakest predictors for both the male and female groups compared to the other indices.

**Table 3:** Diagnostic parameters of the anthropometric indices for predicting T2DM

**Index**	**Sensitivity**	**Specificity**	**Cutoff**	**AUC (95% CI)**	***P*** **value**
**Male**					
BMI	86.40	27.60	25.09	0.573 (0.530, 0.615)	0.010
WHtR	88.00	34.90	0.52	0.648 (0.605, 0.689)	< 0.001
WHR	73.00	53.18	0.91	0.654 (0.612, 0.695)	< 0.001
ABSI	81.00	36.32	0.08	0.599 (0.555, 0.641)	0.001
BRI	88.00	34.91	3.92	0.648 (0.605, 0.689)	< 0.001
BAI	81.00	36.79	27.27	0.590 (0.546, 0.632)	0.002
VAI	64.58	52.97	8.00	0.596 (0.552, 0.640)	< 0.001
**Female**					
BMI	63.08	53.00	28.75	0.610 (0.585, 0.635)	< 0.001
WHtR	73.56	52.80	0.55	0.685 (0.661, 0.709)	< 0.001
WHR	76.56	52.26	0.80	0.687 (0.663, 0.710)	< 0.001
ABSI	51.92	72.71	0.07	0.669 (644, 0.692)	< 0.001
BRI	73.56	52.80	4.34	0.685 (0.661, 0.709)	< 0.001
BAI	40.38	74.69	39.95	0.583 (0.557, 0.608)	0.002
VAI	58.38	66.50	6.15	0.658 (0.633, 0.683)	< 0.001

*BMI* Body mass index, WC Waist circumference, *WHtR* Waist-to-height ratio, *WHR* Waist-to-hip ratio, *ABSI* A body shape index, *BRI* Body boundness index, *BAI* Body adiposity index, *VAI* Visceral adiposity index, *OR* Odds ratio, *AUC* Under the ROC curve, and *CI* Confidence interval

## Discussion

The present study aimed to delineate the relationship between different anthropometric indices and diabetes risk. Our baseline data from the 14-year cohort of FDRs of T2DM patients among Iranian patients revealed that, in both women and men, the BRI, BMI, BAI, WHtR, ABSI, WHR and VAI were significantly greater in the T2DM group than in the non-T2DM group. In women, almost all the indices mentioned above had moderate sensitivity and specificity. However, in men, these indices had high sensitivity but low specificity. The WHR, WHtR, and BRI were the strongest predictors in both men and women, with cutoffs of 0.91, 0.52, and 27.27 in men, respectively, and 0.80, 0.55, and 39.95 in women.

As mentioned before, compared with the other indices, the WHR, WHtR, and BRI were the strongest predictors of T2D risk, while BMI and BAI were the weakest predictors among both the male and female groups. While BMI and the BAI had high sensitivity (86.40 and 81%, respectively), they had relatively low specificity (27.60 and 36.79%, respectively) for predicting T2D risk in men. Even though BMI and the BAI are not good predictors of a diabetes diagnosis in men, these two indices, as well as other indices, have high sensitivity. In other words, all these indices had a relatively low false-positive rate in the diagnosis of diabetes in men, which indicates the capability of these indices to diagnose diabetes. In women, our results showed that the ABSI and BAI, along with the VAI, had relatively moderate specificity. In other words, these patients do not have high false positives, which indicates their ability to diagnose nondiabetic individuals. In total, the three indices WHR, WHtR, and BRI seem to be better at distinguishing diabetic patients from nondiabetic patients.

In the present study, the ABSI index in men was not a good predictor of T2DM risk, which is consistent with the results of Yang. et al. study. However, this index performed well among women. Furthermore, that study revealed BMI to be a stronger predictor of WC, WHtR, VAI, and BRI, which contradicts the results of our study. The different target populations may also explain this difference [4].

Several researchers suggest combining anthropometric indices to better predict T2DM risk [41], while others note increased specificity but decreased sensitivity and positive predictive value when using joint measures [42]. The VAI is calculated using both anthropometric indices (WC and BMI) and laboratory parameters (HDL-C and TG) [23, 40]. This index is positively correlated with visceral adipose tissue and insulin resistance, with its value in predicting T2DM having been shown in both Caucasian [40] and Asian populations [43]. In the present study, we found that the VAI had moderate sensitivity and specificity, indicating that it must be used in combination with the patient’s clinical profile. Furthermore, its AUC was near that of simpler indices, meaning that it may not necessarily be worth evaluating when simpler indices are available. These findings are in line with a similar study on a similar population, which concluded that while the VAI is a robust predictor of T2DM, its predictive power resembles that of BMI, WC, WHtR, and WHR [44]. This concept is also supported by the findings of a large, four-year study on an adult Chinese population [45]. Hence, while the superiority of the VAI over other anthropometric indices has emerged as a common theme in recent years [46], the extent to which it can improve clinical practice is unclear.

In the cohort study of Zafari et al. conducted in Tehran, the derived cutoff values for BMI, WC, WHtR, WHR, and HC were 25.56 kg/m2, 89 cm, 0.52, 0.91, and 96 cm, respectively, in males and 27.12 kg/m2, 87 cm, 0.56, 0.83, and 103 cm, respectively, in females. Among these indices, the WHtR had the greatest discriminatory power [42]. Our study’s cutoff points were slightly different, possibly due to population differences. In Germany, stronger associations were established between indices that reflect abdominal obesity (WC and WHtR) and incident T2DM than between BMI and weight, with WHtR being the strongest predictor [47]; our results are in general agreement with this concept.

A number of similar studies have been conducted on Asian populations. In the Jinchang Cohort Study, Ding et al. reported that the AUC of BMI was greater than that of WC and WHtR in predicting T2DM in Asians. The cutoff points for BMI, WC, and WHtR for predicting T2DM were 24.6 kg/m2, 89.5 cm, and 0.52, respectively, in men and 23.4 kg/m2, 76.5 cm, and 0.47, respectively [12]. Yang et al. reported that BMI, WC, the WHtR, the VAI, and the BRI were positively associated with incident T2DM risk in an elderly Chinese population, with BMI representing the strongest predictor in both men and women (AUC = 0.655 and 0.635, respectively) [4]. Our results suggested a higher cutoff for BMI, in line with the findings of a previous study. In a previous study, the strongest predictor of T2DM incidence was the WHtR in men and BMI in women [48]. BMI has maintained its popularity in the clinic over the years, with strong evidence in favor of its independent link with T2DM [49]. However, in two large cohort studies from the USA, the WHtR performed better than BMI in predicting T2DM [50]. Hence, variations between populations must be considered in clinical decision-making, with the value of indices varying in each population. An interesting prospect is the use of modified indices for each population, for example, the Chinese VAI (CVAI), which performed better than the VAI, BMI, WC, WHR, and WHtR in predicting both prediabetes and T2DM in Chinese adults [51].

The present study has encountered some limitations. Firstly, we used secondary data for this study and did not have control over data collection or the ability to add new information. Another limitation of this study is that due to the unavailability of several indicators, such as ankle and hand circumference or arm circumference, we could not evaluate other new anthropometric indices. Another limitation of our study is that information on postmenopausal women was not available to the researchers. Therefore, further investigations might be required to examine whether menopause and stratification of women based on the menopause status can mediate the association of anthropometric indices and risk of T2DM. The other key limitation of this study was its lack of evaluation of the effects of anthropometric indices on prediabetes, which may be valuable for guiding screening interventions. Nonetheless, the extensive study period and relatively large sample size provided valuable findings. Future studies should focus more on prediabetes to improve screening and prevention rather than disease diagnosis. Population-based modifications to the VAI formula may also be worth exploring.

## Conclusions

The WHtR, BRI, VAI, and WHR outperform the more conventional anthropometric indices in predicting T2DM in FDRs of diabetic patients in this population. Notably, the WHtR, BRI, VAI, and WHR were significantly greater in the T2DM group than in the non-T2DM group. Nonetheless, WHtR and WHR are more practical and relatively simpler to calculate and evaluate, as compared to Visceral Adiposity Index (VAI) and Body Roundness Index (BRI), making them more accessible for healthcare professionals and individuals. Therefore, it is recommended to prioritize the use of WHtR and WHR in T2DM prediction. However, the nuanced sex-specific variations in sensitivity and specificity suggest that a tailored approach may be crucial in clinical applications. These indices, which are finely tuned to capture the intricacies of abdominal obesity and visceral adiposity, have emerged as powerful indicators. Nonetheless, the extent of its superiority in justifying its widespread use in clinical practice remains questionable. In essence, our study not only substantiates the importance of specific anthropometric indices in predicting T2DM risk but also opens the door to a future where personalized risk assessment tools may redefine how we approach preventive strategies.

## Data Availability

The datasets used and/or analyzed during the current study are available from the corresponding author upon reasonable request.

## Abbreviations

FDRs: First-degree relatives
T2DM: Type 2 diabetes mellitus
DM: Diabetes mellitus
BMI: Body mass index
WHO: World Health Organization
WC: Waist circumference
WHtR: Waist-to-height ratio
WHR: Waist-to-hip ratio
ABSI: A Body shape index
BRI: Body roundness index
BAI: Body adiposity index
VAI: Visceral adiposity index
HC: Hip circumference
ROC: Receiver operating characteristic
